# No Difference in Pain and Activities of Daily Living Between Immobilization in External and Internal Rotation Following Acute Anterior Shoulder Dislocation: Results of a Randomized Controlled Trial

**DOI:** 10.7759/cureus.81732

**Published:** 2025-04-04

**Authors:** Deepak Ranjan Patro, Gopisankar Balaji, Sandeep Nema, Raja Vigneswar

**Affiliations:** 1 Orthopaedics, Jawaharlal Institute of Postgraduate Medical Education and Research, Pondicherry, IND; 2 Orthopaedics, All India Institute of Medical Sciences, Raipur, Raipur, IND

**Keywords:** external rotation, immobilization, internal rotation, shoulder dislocation, trauma

## Abstract

Background

The conventional treatment for anterior shoulder dislocations is immobilising the arm in adduction and internal rotation. However, recent basic science and clinical data indicate that immobilization in external rotation can reduce recurrent instability. The use of external rotation brace is not widespread, due to compliance and convenience issues. The purpose of our study is to compare pain and restriction in day-to-day activities experienced by patients after being immobilized in the two types of braces (external rotation and internal rotation) at the end of one week.

Methods

Between February 2023 to July 2024, 50 patients with acute shoulder dislocation (primary or recurrent) were randomized (concealed, computer-generated) to immobilization with either an internal rotation brace (n=26) or an external rotation brace (n=24). Validated scores (Pain-visual analog scale (VAS) for pain, Katz Index for independence in activities of daily living) were used to collect data on the day of Immobilization and at the end of one week of immobilization.

Results

There was no statistically significant difference (P>0.05) in Pain-VAS and Katz Index scores among the two brace types (external rotation and internal rotation) at the end of one week of follow-up. There was no loss in follow-up in either of our study groups.

Conclusion

Immobilization in external rotation after reduction of acute shoulder dislocation did not significantly differ from immobilization in internal rotation in terms of pain and limitations in activities of daily living experienced by the patient in the first week of immobilization.

## Introduction

The shoulder's anatomy makes it prone to dislocations. Anterior dislocations are the most common, accounting for up to 45% of all [1]. Since the Hippocrates era, post-reduction immobilization of the shoulder has been done in adduction and internal rotation (IR) [2]. However, little was known about the best position to heal capsulolabral lesions. Itoi et al.'s studies have shown that immobilization in external rotation (ER) can lead to better healing of capsulolabral lesions than conventional IR [3-13]. This is likely to reduce recurrence rates [14-23]. However, subsequent studies by Liu et al., Paterson et al., and Vavken et al. have not consistently demonstrated a significant reduction in recurrence rates with ER immobilization [24-26]. Despite promising early evidence, no consensus exists on immobilization’s optimal type and duration. The controversy around the efficacy of ER immobilization persists, partly due to compliance issues. Patients often find ER braces uncomfortable and inconvenient, affecting their treatment adherence [27-29]. This is compounded by the lack of prospective trials comparing the effects of ER and IR immobilization on daily activities and pain relief.

This study aims to fill this gap by comparing pain and limitations in daily activities between ER and IR immobilization. We aimed to compare pain, restriction, or limitations in day-to-day activities experienced by patients after being immobilized in the two types of braces (ER and IR) at the end of one week.

## Materials and methods

Study design and setting

This study was a single-centre, parallel-group, open-labelled randomized clinical trial. It was conducted from February 2023 to July 2024 at a level 3 trauma centre in India. The Institute Ethics Committee approved the study (JIP/IEC/2023/01/08) and registered it in the Clinical Trials Registry of India (CTRI/2024/02/062893). Patients 18 years or older, of either gender, presenting with acute anterior shoulder dislocation of less than one week's duration confirmed radiologically undergoing closed reduction were eligible for enrolment in the trial. Participants were eligible for inclusion in the study irrespective of the number of times they dislocated. Shoulder dislocations with associated glenoid /humerus/coracoid/acromion fractures, nerve deficit vascular injury, or any other systemic injury were excluded. The study followed the International Conference on Harmonization Guidelines for Good Clinical Practice and the Declaration of Helsinki. Written informed consent was obtained from all patients.

Patient selection

Participants were eligible for inclusion in the study if they were 18 years or older, of either sex with a radiologically confirmed, recent (less than or at one week) isolated shoulder dislocation without associated fractures of glenoid/humerus/coracoid/acromion, nerve deficit or vascular injury or any other systemic injury.

Randomization and intervention

A randomization sequence was generated from the website sealedenvelope.com. Each new participant's unique identity number was entered into the website, which provided a randomization code that guided the assigning of participants to one of the intervention groups. The allocation was concealed through a sealed opaque envelope, opened after closed reduction to allocate patients into either of the groups.

All participants eligible for the trial were included after the closed reduction of the shoulder. After ruling out other associated injuries, participants were randomly allocated to the IR or ER brace arm.

**Figure 1:**
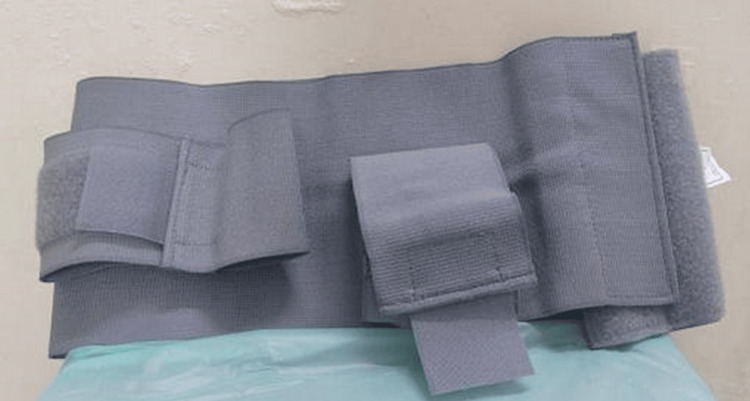
Image showing the commercial internal rotation brace

**Figure 2:**
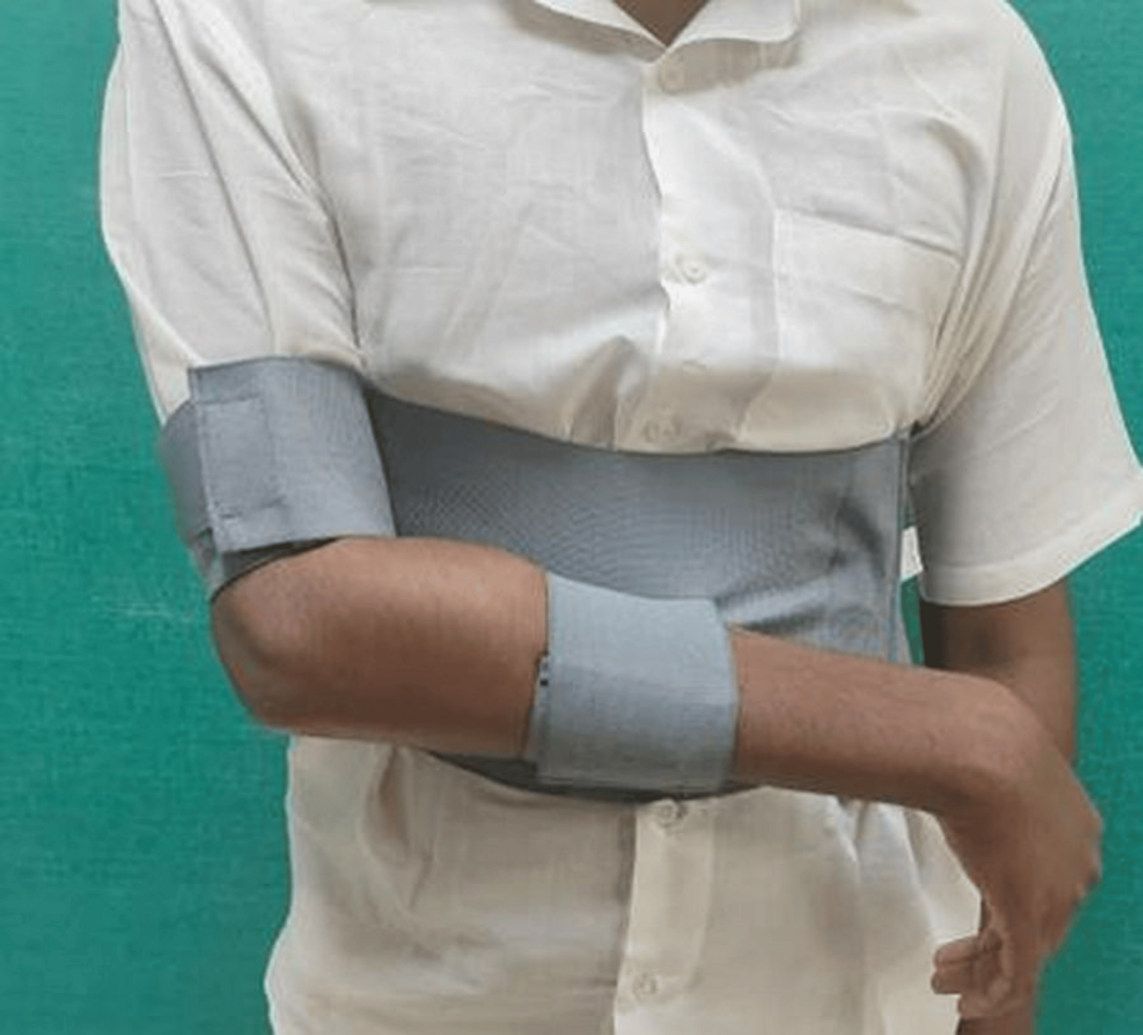
Patient immobilized with a commercial internal rotation brace (front)

**Figure 3:**
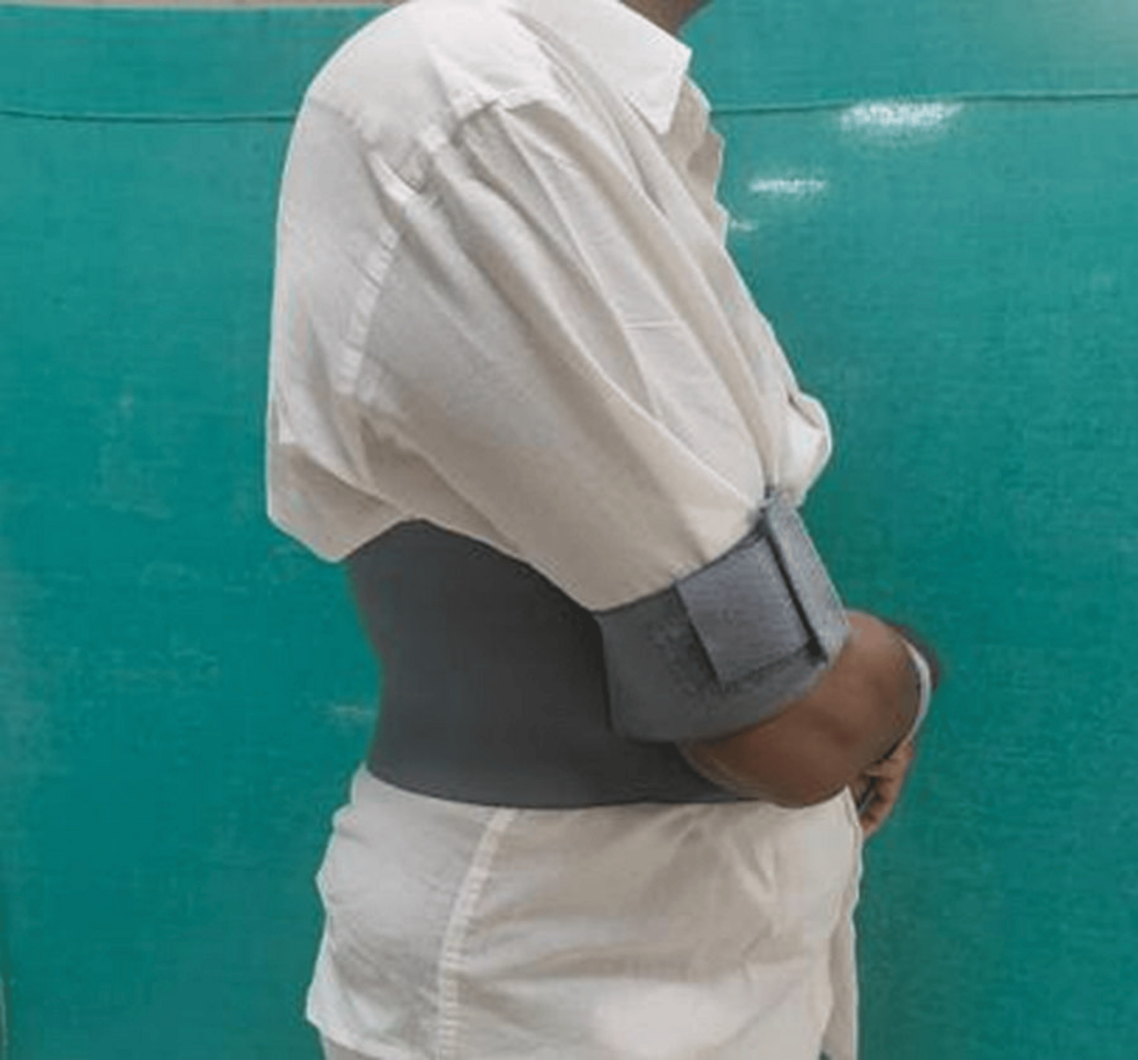
Patient immobilized with a commercial internal rotation brace (side)

In the IR group, commercially available braces immobilizing the shoulder in adduction and internal rotation were used, and participants were discharged (Figures 1-3).

**Figure 4:**
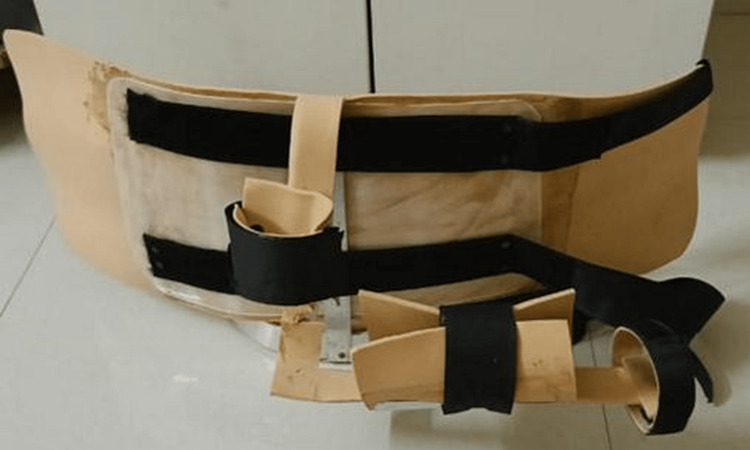
Image showing custom made external rotation brace

**Figure 5:**
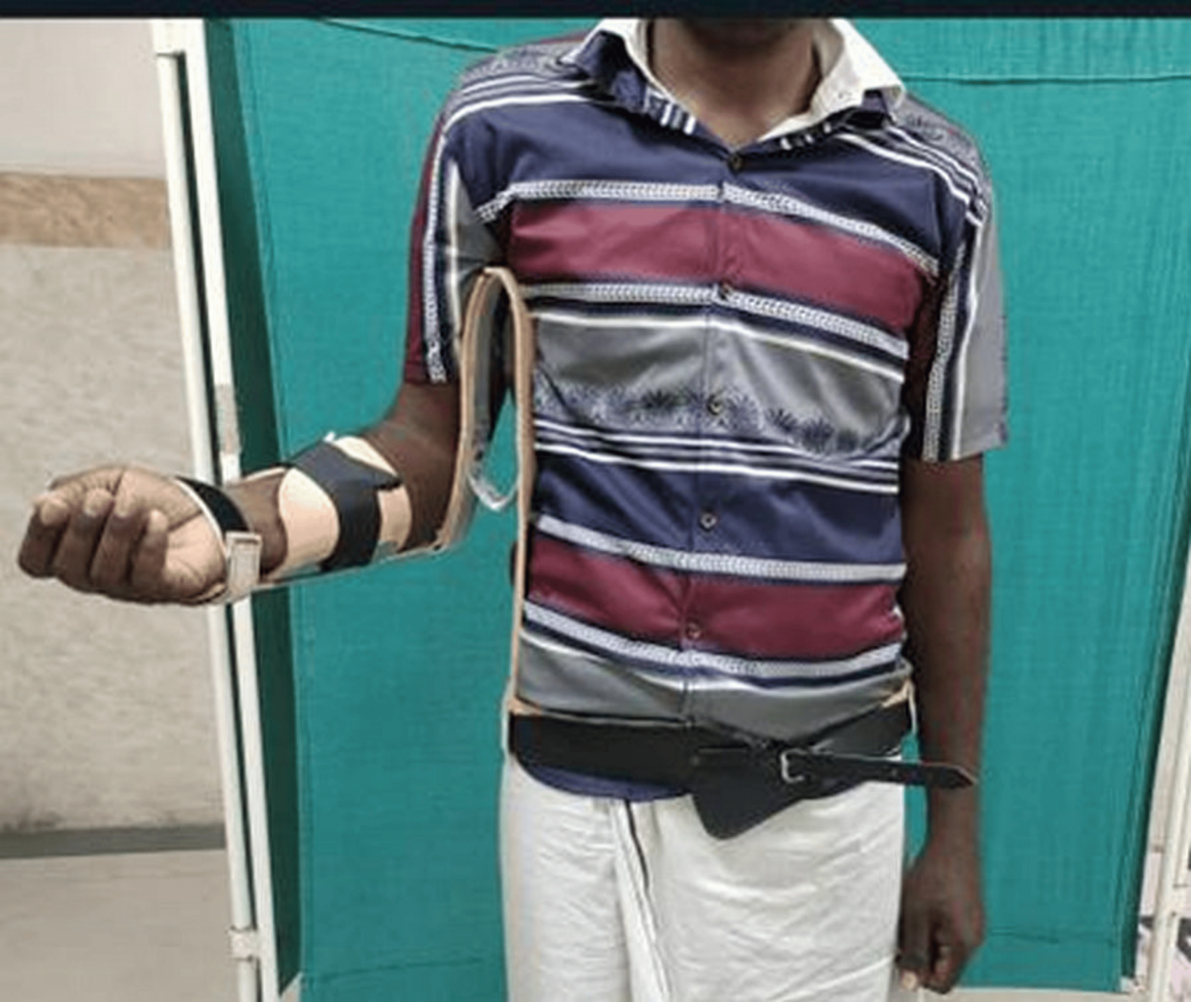
Patient immobilized with custom-made external rotation brace (front)

**Figure 6:**
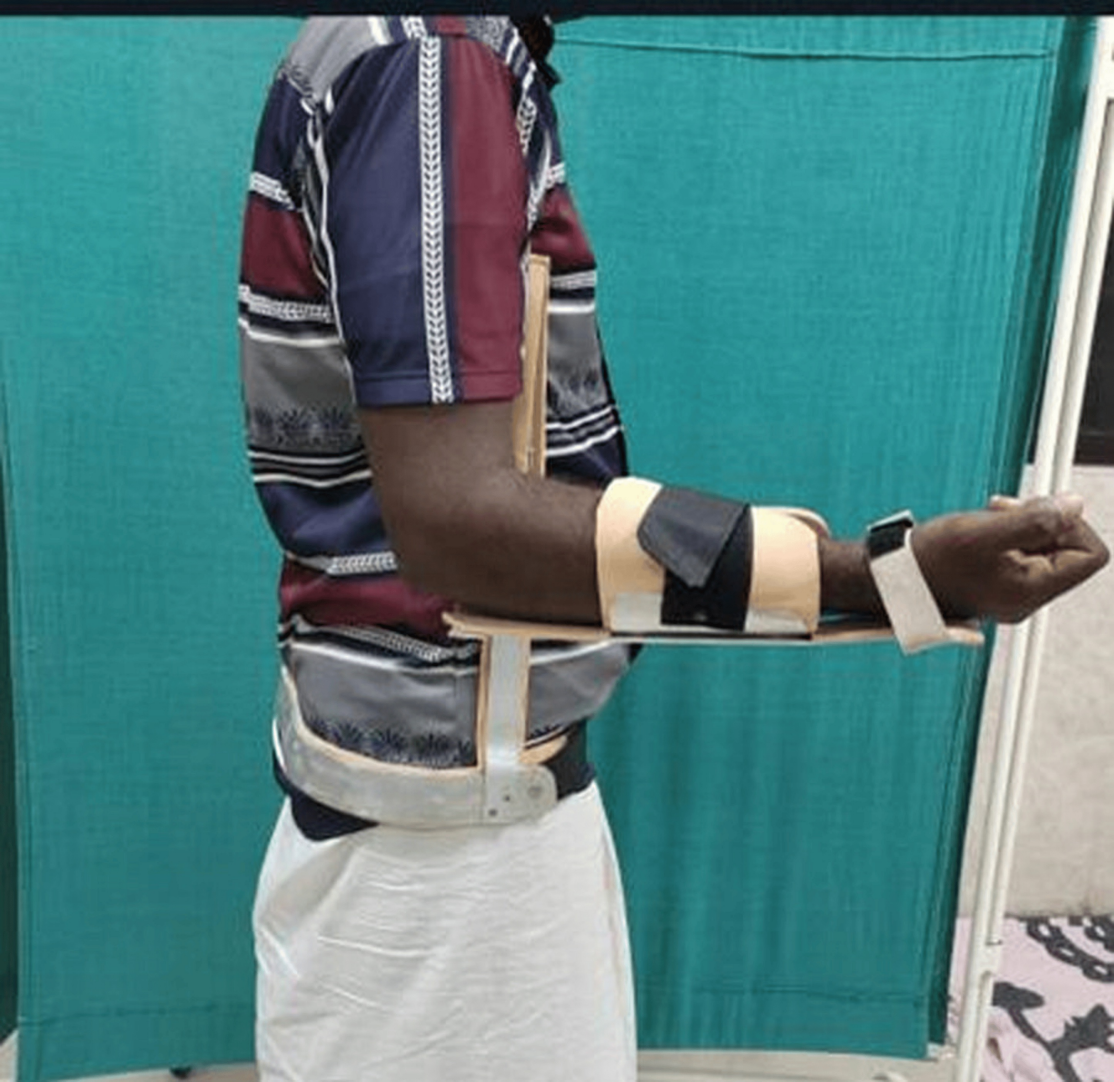
Patient immobilized with custom-made external rotation brace (side)

Pain-VAS score and Katz Index scores were noted at the end of the day. After a week, the patient was followed up in the Orthopaedic OPD to record the above outcome parameters. In the ER group, a custom-made brace from our Orthotic section was used to immobilize the shoulder in 15 degrees of ER with the arm by the side of the body (Figures 4-6).

Post bracing, a similar data collection protocol was followed, as mentioned for the internal rotation bracing arm.

**Figure 7:**
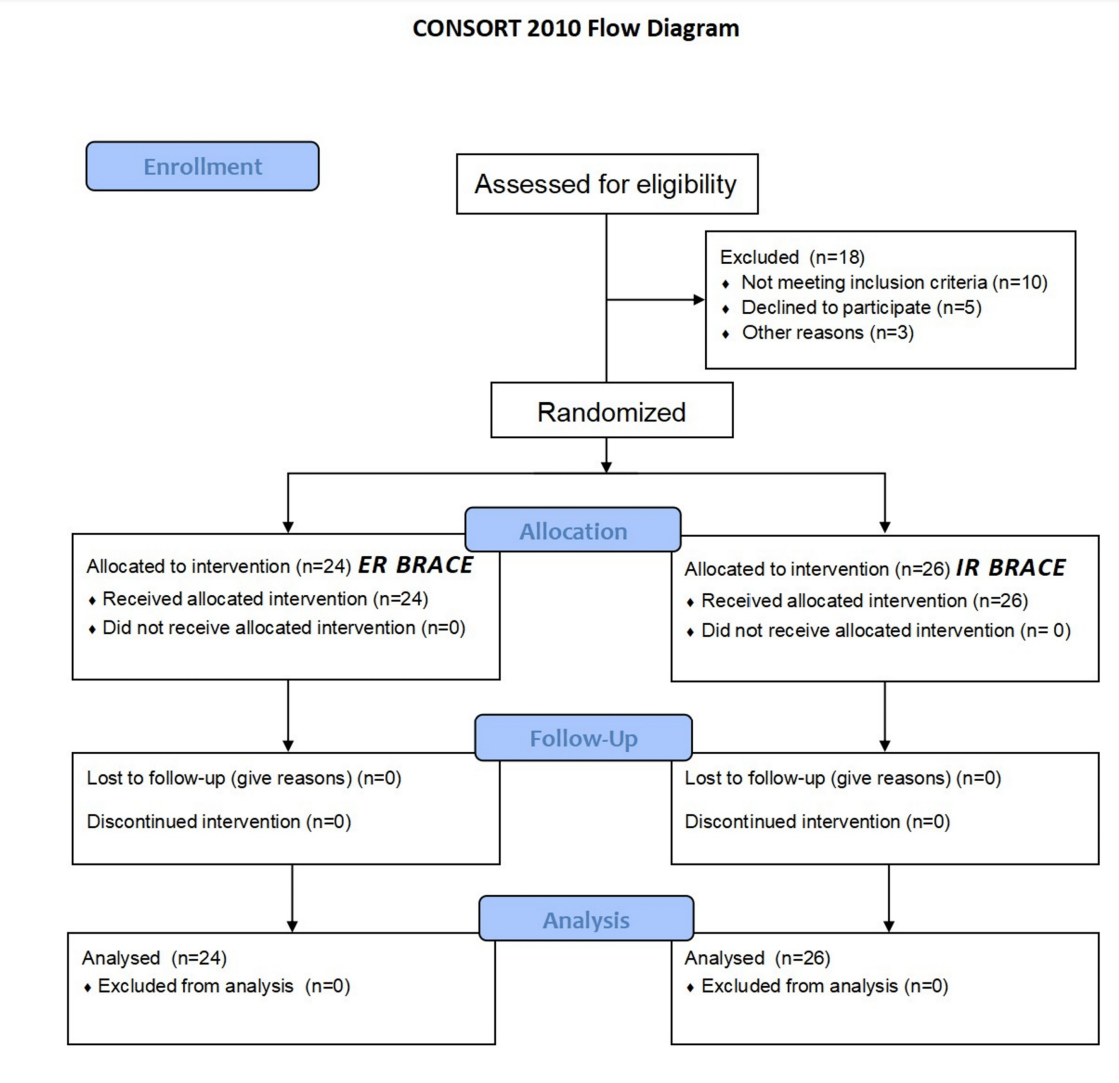
Consolidated Standards of Reporting Trials (CONSORT) flowchart showing the study methodology ER: external rotation, IR: internal rotation

Patients were instructed to wear the brace 24 hours a day (except when taking a shower). If a patient met any adverse event during the trial period, the patient was reviewed on an emergency basis in the Orthopaedic casualty/OPD. The Consolidated Standards of Reporting Trials (CONSORT) flowchart of the methodology is shown in Figure 7.

Outcome measures

Pain measured on VAS after closed reduction and immobilization at one week in either of the groups was the primary outcome variable. It was measured on a scale of 1 to 10.

The secondary outcome measured was the impact on activities of daily living. It was calculated using the Katz Index of independence in activities of daily living. It has six components: (1) bathing, (2) dressing, (3) toileting, (4) transferring, (5) continence, and (6) feeding. One point is given to each component if the patient is independent (with no supervision, direction, or personal assistance) and 0 for dependence (with supervision, direction, personal assistance, or total care).

Statistical analysis

Sample size calculation was done based on the analysis of the post-randomization treatment means (i.e., the POST method). The sample size calculation for the trial assumed that a two-point difference in the mean day one and day seven Pain-VAS scores between the ER and IR groups would be considered a minimum clinically significant difference (MCID). Using a standard deviation of 2.2 for Pain-VAS and Katz scores, a power of 90%, and a two-sided significance of 5%, the sample size was 26 per group.

Demographic data (patients' gender, number of dislocations grouped into two categories (category 1 = less than five dislocations, category 2 = five or more dislocations)) were recorded as nominal variables and were expressed in frequencies. Outcome variables: patient's age, VAS score for pain, and Katz score for independence in activities of daily living were recorded as continuous variables. They were expressed as mean ± standard deviation or median ± 95% confidence interval depending upon the normality of distribution of the data.

The normality of data was tested with Kolmogorov-Smirnov and Shapiro-Wilk tests; in case of any discrepancy between the tests, normality was decided based on the Shapiro-Wilk test. Nominal variables between the two trial groups were compared using chi-square or Fisher's exact test. Normally distributed continuous variables between the two trial groups were compared with an independent t-test. Non-normally distributed continuous variables between the two trial groups were compared using the Mann-Whitney U test. Statistical significance was set at p<0.05 level. 

## Results

Population demographics

We recruited 50 adults treated in the emergency room with anterior shoulder dislocation and randomly assigned them in a 1:1 ratio to receive either an IR or ER brace. The mean age of the participants was 39.8 (SD=17.3) years in the IR group and 36.8 (SD=18.6) years in the ER group, with no statistically significant difference (P=0.492). The male percentage was 96% in the ER brace group and 70% in the IR brace group (Table 1).

**Table 1:** Demographic comparison between the two groups ¶ Mann-Whitney U test * Chi-square test

CHARACTERISTIC	EXTERNAL ROTATION BRACE GROUP (N=24 )	INTERNAL ROTATION SLING GROUP (N=26 )	P VALUE	STATISTICS VALUE
Mean age in years (range)	39.8 (17-74)	36.8 (18-75)	>0.05	Z=0.690¶
Sex (male/female) (number of patients)	17/7	25/1	>0.05	χ²=5.593*
Number of Category 1 dislocation (<5) patients (number of patients)	17	13	>0.05	χ²=2.256*
Number of Category 2 dislocation (≥5) patients (number of patients)	7	13	>0.05	χ²=2.256*
Right-sided dislocation (number of patients)	15	13	>0.05	χ²=0.365*
Left-sided dislocation (number of patients)	9	13	>0.05	χ²=0.365*

Regarding the Pain-VAS and Katz scores, the mean Pain-VAS scores on day one and day seven, indicating pain levels following immobilization, were marginally lower in the ER brace group. Still, the difference did not reach statistical significance (P=0.73 for day one and P=0.17 for day seven). The mean Katz scores, reflecting the ease of day-to-day activities, were slightly higher in the external rotation brace group on days one and seven. Still, the difference did not reach statistical significance (P=0.52 for day one, P=0.16 for the Katz score on day seven) (Tables 2-4).

**Table 2:** Mean Pain-visual analog scale (VAS) and Katz scores in the two groups *  Mann–Whitney test ^ t(df), t=test statistics, df= degree of freedom # Two-sample t test

CHARACTERISTICS	EXTERNAL ROTATION GROUP	INTERNAL ROTATION GROUP	P VALUE	STATISTICS VALUE
PAIN-VAS SCORE AT DAY 1, Mean±SD	4.7±1.4	4.8±0.9	>0.05	z = 0.339^*^
PAIN-VAS SCORE AT DAY 7, Mean±SD	3.7±0.95	4.15±1.2	>0.05	t(48)^^^= 1.3810^#^
KATZ SCORE AT DAY 1, Mean±SD	4.5±0.8	4.3±0.8	>0.05	z = -0.632*
KATZ SCORE AT DAY 7, Mean±SD	4.6±0.6	4.3±0.7	>0.05	t(48)^​​​​​​​^^= -1.4244^#^

**Table 3:** Comparison of Pain-visual analog scale (VAS) and Katz scores among patients with less than five dislocations in the two brace groups * Mann–Whitney test ^ t(df), t=test statistics, df= degree of freedom # Two-sample t test

CHARACTERISTICS	EXTERNAL ROTATION GROUP	INTERNAL ROTATION GROUP	P VALUE	STATISTICS VALUE
PAIN-VAS SCORE AT DAY 1, Mean±SD	3.9±0.9	5.3±1.1	<0.05	Z=3.857^*^
PAIN-VAS SCORE AT DAY 7, Mean±SD	3.4±1.1	4.2±1.0	>0.05	t(48)^^^=2.6003^#^
KATZ SCORE AT DAY 1, Mean±SD	4.5±0.6	4.3±0.9	>0.05	Z=-1.150^*^
KATZ SCORE AT DAY 7, Mean±SD	4.5±0.6	4.4±0.7	>0.05	t(48)^^^=-0.6496^#^

**Table 4:** Comparison of Pain-visual analog scale (VAS) and Katz scores among patients with five or more dislocations in the two brace groups * Mann–Whitney test ^ t(df), t=test statistics, df= degree of freedom # Two-sample t test

CHARACTERISTICS	EXTERNAL ROTATION GROUP	INTERNAL ROTATION GROUP	P VALUE	STATISTICS VALUE
PAIN-VAS SCORE AT DAY1, Mean±SD	4.6±1.2	4.7±1.4	>0.05	Z=0.133^*^
PAIN-VAS SCORE AT DAY7, Mean±SD	3.8±1.2	4±1.2	>0.05	t(39)^^^=0.392^#^
KATZ SCORE AT DAY1, Mean±SD	4.1±1.0	4.3±0.7	>0.05	Z=0.440^*^
KATZ SCORE AT DAY7, Mean±SD	4.3±0.6	4.4±0.6	>0.05	t(39)^^^=0.4951^#^

Further analysis indicated that patients with less than five dislocations and immobilized in the ER experienced lower Pain-VAS scores, indicating better pain relief than those immobilized in the IR group at day one (P<0.05). Similarly, the former group had higher Katz scores, although the difference did not reach statistical significance (P>0.05). On day seven, there was no statistical difference in Pain-VAS and Katz scores in the same group (P>0.05).

The Pain-VAS scores were lower for patients with more than five dislocations and immobilized in the ER, indicating better pain relief. Katz scores were higher, indicating more dependence on day-to-day activities. Still, the differences were not statistically significant (P>0.05).

Our findings also revealed that the dislocation did not affect independence in activities of daily living (P>0.1).

## Discussion

We did not find a statistically significant difference in pain and dependence on day-to-day activities between the groups immobilized in ER and IR on days one and seven. The study addressed the dependence on day-to-day activities between groups immobilized in ER and IR on days one and seven post-treatment. The findings showed that the difference in dependency on day-to-day activities between the two groups was neither clinically relevant nor statistically significant. However, an interesting observation was made regarding pain relief: patients who had experienced less than five dislocations before the study showed better pain relief when immobilized in the ER position. This highlights a potentially significant aspect of post-reduction care following acute shoulder dislocations, as most clinical studies to date have not extensively compared pain relief and its impact on activities of daily living between these two immobilization strategies in the immediate post-reduction period. In this study, patients were immobilized after the reduction of acute shoulder dislocation in a 15-degree ER, based on previous literature by Heidari et al., which suggested that a position of 15-degree abduction and 10-degree ER is safe and conducive for labrum healing to the glenoid, providing a substantial rationale for the chosen immobilization strategy [2,27].

Overall, there was no significant difference between the two groups in terms of Katz score (independence in activities of daily living) [30,31] both on days one and seven, implying patients of both groups had similar difficulty levels in performing daily activities in the first week of immobilization. Zhang et al., in their meta-analysis, showed similar compliance rates among patients immobilized in the above two braces [28]. Our study results fall along similar lines, as pain and independence in day-to-day activities could be significant factors affecting compliance, which were identical in both our study groups. Whelan et al. reported similar compliance rates among immobilization in external and internal rotation [32,33]. In contrast, Itoi et al. and Liavaag et al. reported higher compliance rates in the ER brace than in the IR brace [7,34].

Our study findings contradicted those of the RCT by Chan et al. Despite this, notable differences existed between their study and ours. While they immobilized the shoulder at 30 degrees of each ER and abduction, we maintained the shoulder in neutral abduction and 15 degrees of ER. Their study reported compliance rates, comfort levels, and exercise frequency at three, 12, and 24 months, whereas we recorded pain and dependence in day-to-day activities on days one and seven post-reduction and immobilization. The trial was discontinued by the investigators due to poor recruitment, compliance, and difficulties in outcome follow-up [28].

Additionally, a meta-analysis by Zhang et al. indicated lower complications such as axillary rash and hand pain in the external rotation group compared to the internal rotation group. Nonetheless, in our study, none of the group patients reported any complications [29].

The endpoint of this study focused on assessing pain levels and independence in daily living activities at one week post closed reduction and immobilization for two different patient groups. It was found that the duration of shoulder immobilization - whether up to one week or extending beyond three weeks - did not significantly impact the recurrence rates over periods of two, five, 10, and 25 years [26,35].

A notable strength of the study lies in its design as a randomized controlled trial, characterized by a well-defined and rigorously implemented methodology. This approach effectively minimized potential biases related to chronology, confounding factors, randomization, allocation, and selection. Recruitment was conducted under strict inclusion criteria, and the questionnaires utilized had been previously validated and translated to match the local language context. Importantly, the study experienced no loss to follow-up, enhancing the reliability of its outcomes.

However, the study did also present certain limitations. While immobilization of the shoulder was maintained for three to four weeks post-dislocation, outcome measurements were only conducted on the first and seventh days. Such a schedule may limit the understanding of longer-term effects and recovery progress. Furthermore, the inclusion of patients with varying numbers of dislocation instances without differentiation potentially confounds the results. A more targeted evaluation upon the cessation of brace use - particularly among individuals experiencing their first dislocation - might have provided additional insights into the impacts of our approach.

Compliance with the immobilization regimen, particularly in emergency room settings, was not reported due to the timing of evaluations. As such, the study does not address potential variations in patient adherence to prescribed immobilization.

Another point of investigation was the perceived discomfort associated with ER braces, which anecdotally leads to less frequent prescription. Our findings, however, did not support the assumption that ER braces are significantly less comfortable or acceptable to patients compared to other forms of immobilization.

## Conclusions

In conclusion, while the study contributes valuable data to the field, particularly with its robust methodological foundation, it also highlights the need for further research. Future studies are encouraged to delve deeper into aspects of patient acceptability, adherence to treatment regimens, recurrence rates, and overall functional outcomes in the long term.
